# Correction: Analyzing cold hardiness (Based on DTA) of one-year-old branches of peaches

**DOI:** 10.1371/journal.pone.0329836

**Published:** 2025-08-05

**Authors:** Yonghong Li, Jie Li, Zhaoyuan Wang, Guojian Liu, Yu Wang, Ruifeng Chang, Hu Chen, Qihang Tian, Xiaodi Wang

There are errors in the author affiliations. The correct affiliations are as follows:

Yonghong Li^2^, Jie Li^2^, Zhaoyuan Wang^2^, Guojian Liu^2^, Yu Wang^2^, Ruifeng Chang^2^, Hu Chen^2^, Qihang Tian^2^, Xiaodi Wang^1^

**1** Research Institute of Pomology of CAAS, Xingcheng, Liaoning, China, **2** Changli Fruit Institute, Hebei Academy of Agriculture and Forestry Sciences, Changli, Hebei, China.
