# Supplementary material for: Analyzing cold hardiness (Based on DTA) of one-year-old branches of peaches
Source: PLoS One. 2024 Sep 17;19(9):e0306914. doi: 10.1371/journal.pone.0306914 (PMC11407669; doi:10.1371/journal.pone.0306914)
Supplement: Attachment — Submitted filename: Review comments.docx [file pone.0306914.s001.docx]

In this study, an investigation on the low-temperature exothermic (LTE) behavior of one-year-old (1a) branches from sixteen peach cultivars was conducted using a differential thermal analysis (DTA) procedure. The cold hardiness of branches was evaluated by establishing a temperature-injury (LT-I) regression functions of branch xylem. The cold hardiness of 16 peach varieties was compared using the subordinate function value method. Furthermore, the data of browning rate (BR) and electrolyte leakage (EI) from traditional low-temperature freezing analysis were measured and found to be significantly correlated with the degree of xylem damage in LT-I analysis. The research results confirm that LTE analysis is a simple, accurate, and practical method for identifying the cold hardiness of peach branches. This research result is of great significance for the identification of cold hardiness of fruit trees. However, some errors and problems exist in the manuscript need be explained and modified before it is considered for acceptance.

**Comments**

The authors selected Wangjiazhuangmaotao No.2, Zhongnongjinhui, and Chunmei as three representative varieties with strong, moderate, and weak cold hardiness ranks to determine EI and BR. However, Table 3 showed that Zhongnongjinhui and Chunmei are both medium cold-hardiness (MH) variety. In the subsequent results, there was a difference in data of BR between Zhongnongjinhui and Chunmei, both of which were MH variety. Does this indicat that the identification of cold hardness through LTE investment of is not as precise? Please explain in detail.

**Minor points**

1. Please review the manuscript for punctuation errors throughout the text, e.g. line 88 "... electrical leakage (EI)]".
2. Please review the manuscript for the consistency of font size, e.g. line 96 "A total of …", and line 164 "…hardiness…".
3. Please carefully check the reference format and ensure consistency, e.g. line 346 "Am. J. Enol. Vitic", and line 352 "Annals of Botany".
