# Supplementary material for: Analyzing cold hardiness (Based on DTA) of one-year-old branches of peaches
Source: PLoS One. 2024 Sep 17;19(9):e0306914. doi: 10.1371/journal.pone.0306914 (PMC11407669; doi:10.1371/journal.pone.0306914)
Supplement: Attachment — Submitted filename: Response to Reviewers.docx [file pone.0306914.s002.docx]

**Response to the comments of reviewers**

**Manuscript Number**: PONE-D-23-42629

**Manuscript Title:** Analyzing Cold Hardiness (Based on DTA) of One-Year-Old Branches of Peaches

Dear Editors:

Thanks for your kind comments. We have carefully read the whole manuscript and revised it in accordance with the academic editor and the reviewers’ comments. The corrections and additions are highlighted in red color in manuscript. We list the reply as follows:

Guanfu Fu, Ph.D

**Academic Editor**

1. When submitting your revision, we need you to address these additional requirements.

Please ensure that your manuscript meets PLOS ONE's style requirements, including those for file naming. The PLOS ONE style templates can be found at

https://journals.plos.org/plosone/s/file?id=wjVg/PLOSOne_formatting_sample_main_body.pdf and

<https://journals.plos.org/plosone/s/file?id=ba62/PLOSOne_formatting_sample_title_authors_affiliations.pdf>

Reply:We have revised the manuscript according to PLOS ONE's style requirements.

2. In your Methods section, please provide additional information regarding the permits you obtained for the work. Please ensure you have included the full name of the authority that approved the field site access and, if no permits were required, a brief statement explaining why.

Reply:

No permits statement

We are a non-profit scientific research institutions and do not need to provide a permit.

3. Thank you for stating in your Funding Statement:

This work was supported by Key Laboratory of Biology and Genetic Improvement of Horticultural Crops (Germplasm Resources Utilization), Ministry of Agriculture,P.R.China (No. NYZS202305) and Technology Innovation Special Project of Hebei Academy (No. 2022KJCXZX-CGS-6), Key R&D Projects of Hebei Province (No. 21326310D), Modern Agricultural Industrial Technology System of Hebei Province (No. HBCT2023130205) and China Agriculture Research System of MOF and MARA (No. CARS-30-Z-02).

Please provide an amended statement that declares all the funding or sources of support (whether external or internal to your organization) received during this study, as detailed online in our guide for authors at http://journals.plos.org/plosone/s/submit-now. Please also include the statement “There was no additional external funding received for this study.” in your updated Funding Statement.

Please include your amended Funding Statement within your cover letter. We will change the online submission form on your behalf.

Reply:We have submitted our amended Funding Statement in cover letter.

4. Thank you for stating the following in the Acknowledgments Section of your manuscript:

This work was supported by Key Laboratory of Biology and Genetic Improvement of Horticultural Crops (Germplasm Resources Utilization), Ministry of Agriculture,P.R.China (No. NYZS202305) and Technology Innovation Special Project of Hebei Academy (No. 2022KJCXZX-CGS-6), Key R&D Projects of Hebei Province (No. 21326310D), Modern Agricultural Industrial Technology System of Hebei Province (No. HBCT2023130205) and China Agriculture Research System of MOF and MARA (No. CARS-30-Z-02).

We note that you have provided funding information that is not currently declared in your Funding Statement. However, funding information should not appear in the Acknowledgments section or other areas of your manuscript. We will only publish funding information present in the Funding Statement section of the online submission form.

Please remove any funding-related text from the manuscript and let us know how you would like to update your Funding Statement. Currently, your Funding Statement reads as follows:

This work was supported by Key Laboratory of Biology and Genetic Improvement of Horticultural Crops (Germplasm Resources Utilization), Ministry of Agriculture,P.R.China (No. NYZS202305) and Technology Innovation Special Project of Hebei Academy (No. 2022KJCXZX-CGS-6), Key R&D Projects of Hebei Province (No. 21326310D), Modern Agricultural Industrial Technology System of Hebei Province (No. HBCT2023130205) and China Agriculture Research System of MOF and MARA (No. CARS-30-Z-02).

Please include your amended statements within your cover letter; we will change the online submission form on your behalf.

Reply:We have deleted and changed the declaration in the Acknowledgments Section of my manuscript.

5. We note that your Data Availability Statement is currently as follows: All relevant data are within the manuscript and its Supporting Information files

Please confirm at this time whether or not your submission contains all raw data required to replicate the results of your study. Authors must share the “minimal data set” for their submission. PLOS defines the minimal data set to consist of the data required to replicate all study findings reported in the article, as well as related metadata and methods (https://journals.plos.org/plosone/s/data-availability#loc-minimal-data-set-definition).

For example, authors should submit the following data:

- The values behind the means, standard deviations and other measures reported;

- The values used to build graphs;

- The points extracted from images for analysis.

Authors do not need to submit their entire data set if only a portion of the data was used in the reported study.

If your submission does not contain these data, please either upload them as Supporting Information files or deposit them to a stable, public repository and provide us with the relevant URLs, DOIs, or accession numbers. For a list of recommended repositories, please see https://journals.plos.org/plosone/s/recommended-repositories.

If there are ethical or legal restrictions on sharing a de-identified data set, please explain them in detail (e.g., data contain potentially sensitive information, data are owned by a third-party organization, etc.) and who has imposed them (e.g., an ethics committee). Please also provide contact information for a data access committee, ethics committee, or other institutional body to which data requests may be sent. If data are owned by a third party, please indicate how others may request data access.

Reply:Our submission have contained all raw data required to replicate the results of study.

6. When completing the data availability statement of the submission form, you indicated that you will make your data available on acceptance. We strongly recommend all authors decide on a data sharing plan before acceptance, as the process can be lengthy and hold up publication timelines. Please note that, though access restrictions are acceptable now, your entire data will need to be made freely accessible if your manuscript is accepted for publication. This policy applies to all data except where public deposition would breach compliance with the protocol approved by your research ethics board. If you are unable to adhere to our open data policy, please kindly revise your statement to explain your reasoning and we will seek the editor's input on an exemption. Please be assured that, once you have provided your new statement, the assessment of your exemption will not hold up the peer review process.

Reply:I accept your suggestion that all authors decide on a data sharing plan before acceptance.

7. PLOS requires an ORCID iD for the corresponding author in Editorial Manager on papers submitted after December 6th, 2016. Please ensure that you have an ORCID iD and that it is validated in Editorial Manager. To do this, go to ‘Update my Information’ (in the upper left-hand corner of the main menu), and click on the Fetch/Validate link next to the ORCID field. This will take you to the ORCID site and allow you to create a new iD or authenticate a pre-existing iD in Editorial Manager. Please see the following video for instructions on linking an ORCID iD to your Editorial Manager account: https://www.youtube.com/watch?v=_xcclfuvtxQ

Reply:I have ensured that I have an ORCID iD.

**Reviewer #1:**

1. The authors selected Wangjiazhuangmaotao No.2, Zhongnongjinhui, and Chunmei as three representative varieties with strong, moderate, and weak cold hardiness ranks to determine EI and BR. However, Table 3 showed that Zhongnongjinhui and Chunmei are both medium cold-hardiness (MH) variety. In the subsequent results, there was a difference in data of BR between Zhongnongjinhui and Chunmei, both of which were MH variety. Does this indicat that the identification of cold hardness through LTE investment of is not as precise? Please explain in detail.

Reply:The definition of medium cold-hardiness (MH) variety is relatively broad, which can also be divided into three levels: high, medium, and low, so there may be differences in the EI and BR of Zhongnongjinhui and Chunmei.

2. Minor points:Please review the manuscript for punctuation errors throughout the text, e.g. line 88 "... electrical leakage (EI)]".

Reply:We have checked and corrected the the manuscript for punctuation.

We have corrected line 88 "... electrical leakage (EI)]".

3. Minor points:Please review the manuscript for the consistency of font size, e.g. line 96 "A total of …", and line 164 "…hardiness…".

Reply:We have reviewed and corrected the manuscript for the consistency of font size.

4. Minor points:Please carefully check the reference format and ensure consistency, e.g. line 346 "Am. J. Enol. Vitic", and line 352 "Annals of Botany".

Reply:We have checked and corrected the reference format and ensure consistency,e.g. line 346 "Am. J. Enol. Vitic", and line 352 "Annals of Botany", as well as others.

**Reviewer #2:**

1. There are multiple formatting errors throughout the manuscript, such as the incorrect use of spaces in lines 34, 66, 74, 77, etc.

Reply:We have checked and corrected formatting errors formatting errors, such as the incorrect use of spaces in lines 34, 66, 74, 77, etc.

2. Line 80: "the LTE determination method involved cutting" should not be formatted in bold.

Reply:We have checked and corrected formatting errors formatting errors.

3. Fig 1: Graphing error, there is no point C in the diagram.

Reply:We have checked and corrected the annotation error for point C in Fig 1.

4. Line 140: “There” should be “there”.

Reply:“There” is correct writing, and we have corrected the punctuation error before it. 5. Line 256: An “of” should be removed.

Reply:We have checked and corrected the error.

5. Line 260: “wiht” should be “with”.

Reply:We have checked and corrected the error.

7. Line 278: “siince” should be “since”.

Reply:We have checked and corrected the error.

6. PLOS authors have the option to publish the peer review history of their article (what does this mean?). If published, this will include your full peer review and any attached files.

Reply:Authors agrees to option to publish the peer review history.
