# Supplementary material for: Analyzing cold hardiness (Based on DTA) of one-year-old branches of peaches
Source: PLoS One. 2024 Sep 17;19(9):e0306914. doi: 10.1371/journal.pone.0306914 (PMC11407669; doi:10.1371/journal.pone.0306914)
Supplement: Attachment — Submitted filename: Response to Reviewers.docx [file pone.0306914.s003.docx]

Date: June 22, 2024

Dear Editor,

On behalf of my co-authors, we thank you for giving us a chance to revise and improve the quality of our article (PONE-D-23-42629).

We have read the reviewers’ and your comments carefully and have made revision which marked in red in the paper. We have tried our best to revise our manuscript according to the comments. Attached please find the revised version, which we would like to submit for your kind consideration. Here, we would like to explain the changes briefly as follows:

We have carefully checked the reference list to ensure it is complete and correct. And about‘5. Is the manuscript presented in an intelligible fashion and written in standard English?’, We have found an English native speaker with a research background to review our manuscript during revision. And if you think there is any problem, you can raise it at any time. we will look for professional organizations to improve the language.

And about Golden honey No. 1 and Huangjinmi No.1 problem mentioned by the third reviewer, we have altered to use a Golden honey No. 1.

We have written a point-by-point response letter for two reviewers, you can see the details at the end of this letter. To make the reply more visible, Q represents questions raised by reviewers, and A are our answers for these questions. In all, we found these comments are quite helpful. And special thanks to you and reviewers for your good comments again.

I wish this revision will be acceptable for publication in your journal.

Thank you for your consideration. I am looking forward to hearing from you.

Yours Sincerely,

Qihang Tian

**Academic Editor**

**Journal Requirements:**

**Q:**Please review your reference list to ensure that it is complete and correct. If you have cited papers that have been retracted, please include the rationale for doing so in the manuscript text, or remove these references and replace them with relevant current references. Any changes to the reference list should be mentioned in the rebuttal letter that accompanies your revised manuscript. If you need to cite a retracted article, indicate the article’s retracted status in the References list and also include a citation and full reference for the retraction notice.

**A:**We have carefully checked the reference list to ensure it is complete and correct.

**Responses to Editor：**

**Reviewers' comments:**

**Responses to Reviewers：I will no longer explain the affirmative response given by the reviewers, but I will provide my answers to the No and No responses given by the reviewers.**

**Q:**5. Is the manuscript presented in an intelligible fashion and written in standard English?

PLOS ONE does not copyedit accepted manuscripts, so the language in submitted articles must be clear, correct, and unambiguous. Any typographical or grammatical errors should be corrected at revision, so please note any specific errors here.

Reviewer #1: No

Reviewer #3: (No Response)

**A:**We have found an English native speaker with a research background to review our manuscript during revision. And if you think there is any problem, you can raise it at any time. we will look for professional organizations to improve the language

**Q:**6. Review Comments to the Author

Please use the space provided to explain your answers to the questions above. You may also include additional comments for the author, including concerns about dual publication, research ethics, or publication ethics. (Please upload your review as an attachment if it exceeds 20,000 characters)

Reviewer #1: (No Response)

Reviewer #3: The revised manuscript provided by Li et al. has revised and respond correctly to comments from both former reviewers.

However, there is one more comment need to be amended. I found that the authors mentioned “Huangjinmi No.1” in the Abstract section of the manuscript, but I cannot find any related information about “Huangjinmi No.1” in the other sections of the manuscript. So please double check whether this is a typo of “Golden honey No. 1” (as I have read it in the Results section but not mentioned in the Abstract section) and amend the fault. I suggest the manuscript could be accepted after this fault being fixed.

**A:**About Golden honey No. 1 and Huangjinmi No.1 problem mentioned by the third reviewer, we have altered to use a Golden honey No. 1. (see abstract）

**Q:**While revising your submission, please upload your figure files to the Preflight Analysis and Conversion Engine (PACE) digital diagnostic tool, https://pacev2.apexcovantage.com/. PACE helps ensure that figures meet PLOS requirements. To use PACE, you must first register as a user. Registration is free. Then, login and navigate to the UPLOAD tab, where you will find detailed instructions on how to use the tool. If you encounter any issues or have any questions when using PACE, please email PLOS at <a href="mailto:figures@plos.org">figures@plos.org. Please note that Supporting Information files do not need this step.

**A:**We have uploaded figure files to the Preflight Analysis and Conversion Engine (PACE) digital diagnostic tool.
